# An Evaluation of the Effectiveness of Melatonin and n-Acetylcysteine in Cerebral Ischemia-Reperfusion Injury in Adult Rats

**DOI:** 10.3390/medicina59112026

**Published:** 2023-11-17

**Authors:** Hilal Aydin, Ozgur Bulmus, Oguzhan Korkut, Eren Altun, Ali Engin Ulusal

**Affiliations:** 1 Department of Pediatric Neurology, Faculty of Medicine, Balikesir University, Balikesir 10145, Turkey; 2 Department of Physiology, Faculty of Medicine, Balikesir University, Balikesir 10145, Turkey; ozgur.bulmus@balikesir.edu.tr; 3 Department of Medical Pharmacology, Faculty of Medicine, Balikesir University, Balikesir 10145, Turkey; oguzhankorkut@balikesir.edu.tr; 4 Department of Medical Pathology, Health Sciences University, Istanbul Bağcılar Training and Research Hospital, Balikesir 10145, Turkey; erenaltun@hotmail.com; 5 Department of Orthopedics and Traumatology, Faculty of Medicine, Balikesir University, Balikesir 10145, Turkey; aeulusal@gmail.com

**Keywords:** melatonin, middle cerebral artery occlusion, ischemia-reperfusion, n-acetylcysteine

## Abstract

*Background and Objectives:* The purpose of this study was to apply histopathological and immunohistochemical methods to compare the protective efficacy of melatonin and N-acetylcysteine (NAC) application in rats with experimental brain ischemia/reperfusion (I/R) injury induced through occlusion of the middle cerebral artery (MCA), and to evaluate the protective effect of their combined use. *Materials and Methods:* Forty-one young adult male Wistar albino rats were divided into five groups—control (*n* = 8), I/R group (*n* = 8), melatonin (*n* = 8), NAC (*n* = 8), and melatonin + NAC (*n* = 9). *Results:* All scores differed between the groups, apart from vascular congestion (*p* < 0.05). At two-way comparisons, all histological scores were significantly higher in the I/R group than in the control group (*p* < 0.05). No change occurred in the vascular congestion scores with the administration of melatonin, although decreases were determined in all other scores. These decreases were statistically significant for cellular eosinophilic pyknotic degeneration, vacuolization, and edema (*p* < 0.05). All histopathological scores in the group administered NAC together with melatonin were significantly lower than in the I/R group (*p* < 0.05). *Conclusions:* The combined use of NAC and melatonin, the neuroprotective efficacy of which on histopathological parameters is shown in this study, now needs to be supported by further research.

## 1. Introduction

Ischemia is a pathological process that emerges due to a decrease in blood flow to tissues, the prolongation of which causes cellular dysfunction and damage resulting from tissues being deprived of the oxygen and nutrients they require. Subsequent restoration of the blood supply to the ischemic region (reperfusion) is unable to completely reverse this cellular injury, and can even give rise to secondary damage (ischemia-reperfusion [I/R] injury) [1,2,3]. I/R injury is a state encountered in numerous medical fields and has been the subject of research in several organs [4]. Although the pathophysiology of the injury has not been entirely elucidated, numerous cellular and humoral events, such as impaired cellular energy consumption and cell membrane functions, free oxygen radical injury, vasoregulation and endothelial function disorder, and inflammatory response associated with complementary system activation are known to be involved [5,6]. Depending on the duration and severity of ischemia, I/R injury can lead to apoptosis at the cellular level, necrosis, and eventually organ function disturbance and organ failure [7]. Although researchers have tested various different drugs targeting various stages in the pathophysiological process, no effective and safe therapeutic method has yet been developed [8].

Melatonin is an endogenous indolamine produced in the pineal gland from the essential amino acid tryptophan. Production is regulated in a circadian manner by the suprachiasmatic nucleus in association with dark:light cycles [9]. It is also produced at lower levels in the liver, gut, bone marrow cells, lymphocytes, muscle, spleen, thymus, heart, intestine, and epithelial cells [10]. Studies have shown that melatonin exhibits anti-inflammatory, immunomodulatory, antioxidant, and free oxygen radical-scavenging effects, and it has been shown to prevent I/R injury and to exhibit a neuroprotective effect in experimental animal models [11,12,13].

The glutathione precursor N-acetylcysteine (NAC) is a drug derived from the sulfhydryl group. NAC is a precursor of L-cysteine and is widely employed in mucolytic therapy. It is directly converted to metabolites capable of stimulating glutathione synthesis by acting as a free radical scavenger [14,15,16]. Studies have shown that, with its antioxidant effect, NAC reduces I/R injury in the brain [17]. Histopathologically, the groups in the present study were compared in terms of cellular eosinophilic pyknotic degeneration, vascular congestion, vacuolization, necrosis (infarct), and edema scores.

## 2. Materials and Methods

### 2.1. Animals

This study was performed at the Balikesir University Experimental Animals Production, Care, and Research Center, Türkiye, following receipt of approval from the Balikesir University Medical Faculty ethical committee (decision no. 2020/5-1 dated 20 August 2020). Forty-one young adult male Wistar albino rats aged 8–9 weeks and weighing 200–250 g were housed at a temperature of 22–25 °C under diurnal lighting conditions (12-h dark:12-h light). Access to water was permitted, but the animals were fasted for 12 h before the experiment. All experimental procedures were carried out according to the protocols approved by the Institutional Animal Care and Use Committee of Balikesir University.

### 2.2. Experimental Protocol

The experimental animals were divided into five groups—control (*n* = 8), MCA occlusion (MCAO) (*n* = 8) (I/R group), melatonin (*n* = 8), NAC (*n* = 8), and melatonin + NAC (*n* = 9). The experimental protocol commenced once the animals had been anesthetized with intraperitoneal ketamine hydrochloride 75 mg/kg (Ketalar, Parke-Davis, Detroit, MI, USA) and xylazine 8 mg/kg (Rompun, Bayer, Leverkusen, Germany). The animals were randomly assigned into the experimental groups. All experimental procedures were conducted by working blind.

In the control group, a scalpel was used to make an incision from the petrous bone toward the scapula, after which the neck muscles were dissected. The right main carotid arteries were exposed, and the dissection areas were closed with (6/0) silk sutures without occlusion. The wound sites were cleansed with antiseptic solution. In the MCAO group, after the dissection of the neck muscles, the carotid bifurcation around the vagus nerve and the glomus caroticum were dissected. The occipital artery leaving the external carotid artery (ECA) was ligated close to the carotid bifurcation. The internal carotid artery (ICA) was carefully dissected as close as possible to the entrance to the skull. (3/0) Nylon sutures were placed in the ICA through the right ECA and intracranially advanced approximately 17–20 mm from the bifurcation of the carotid communis artery in order to block the entrance to the MCA [18]. After one hour, reperfusion was performed by withdrawing the nylon sutures. Following the closure of the dissection region, the wound site was cleansed with antiseptic solution. In the medication groups, melatonin (15 mg/kg), NAC (50 mg/kg), and melatonin (15 mg/kg) + NAC (50 mg/kg) were intraperitoneally administered 30 min before MCAO. No drugs were applied to the control or I/R groups. However, the I/R group was administered the melatonin solvent ethanol/physiological saline via the intraperitoneal route before the procedure at the same concentration and volume as in the drug group (Table 1).

**Scheme 1:**
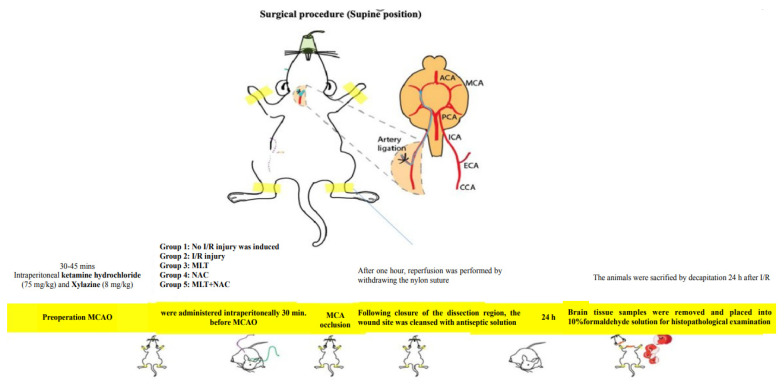
Preparation stages for the MCAO model.

The animals were sacrificed by decapitation 24 h after I/R. Brain tissue samples were removed and placed into 10% formaldehyde solution for histopathological examination (Scheme 1).

### 2.3. Chemicals Employed in the Experiments

Melatonin and NAC (both obtained from Sigma Chemical, Saint Louis, MO, USA) were used during the experiments. Physiological saline was employed as an NAC solvent. Melatonin was dissolved in ethanol and diluted with physiological saline as recommended by the manufacturer.

### 2.4. Histopathological and Immunohistochemical Analysis

The brain tissues were fixed in 10% formaldehyde solution for 48 h and were then subjected to macroscopic examination. The cerebral cortex tissues were used for evaluation. The tissues were horizontally divided into two halves, after which sampling was performed including the entire medullary and cortical layers. The specimens were subjected to routine procedures with alcohol and xylene series and embedded in paraffin blocks. At least four 3-µm sections taken from the blocks using a microtome (Leica 2245, Nussloch, Germany) were placed onto two glass slides. These sections were histochemically stained with hematoxylin and eosin (H&E) and crystal violet. The preparates obtained during the histochemical and immunohistochemical procedures were evaluated by a blinded pathologist under a light microscope (Nikon, EclipseCi, Tokyo, Japan).

At histopathological examination, cellular eosinophilic pyknotic degeneration, vascular congestion, vacuolization, necrosis (infarct), and edema parameters [scored in four categories based on the intensity of alterations: 0, absent; 1, mild; 2, moderate; or 3, severe] were assessed in cerebral cortex tissues. The cerebral cortex of the rats in the control group was used as normal control tissue.

Immunohistochemical analyses for the evaluation of apoptosis in tissue specimens were performed with the TUNEL test [19]. Other specimens separately collected were stained for the performance of that test. Cells heading to apoptosis were identified using an ABP Biosciences TUNEL chromogenic Apoptosis Detection Kit (Catalog No. A049, Beltsville, MD, USA) in line with the manufacturer’s instructions. At least 100 normal and apoptotic cells were counted in randomly selected areas of the sections at ×10 magnification. Histological scoring was also performed in terms of neuronal changes in the cortex region in both hemispheres. The Apoptotic Index (AI) was calculated as the proportion of apoptotic cells to total cells (normal + apoptotic). Values of 0–5% were regarded as low and values of 6% or above as high.

### 2.5. Statistical Analysis

All statistical analyses were carried out on SPSS statistical software (SPSS for Windows, version 11.0). All data were presented as mean plus standard deviation (SD). The normal distribution suitability of the data was evaluated with the Shapiro-Wilk test. Differences in measured parameters among the groups were analyzed using the Kruskal–Wallis test. Dual comparisons between groups exhibiting significant values were evaluated with the Mann–Whitney U-test. These differences were considered significant at values less than 0.05.

## 3. Results

**Figure 1:**
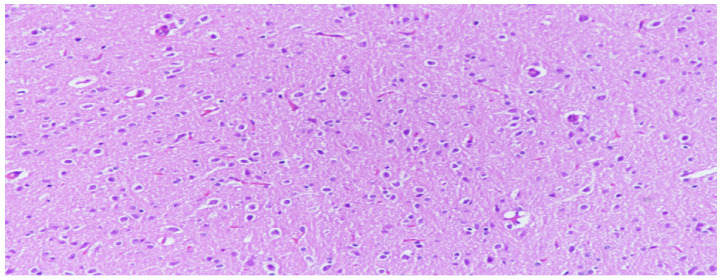
Neuron cells with normal structures were characterized by clear edges and evenly stained nuclei and evenly stained intercellular substance, (H&E, 200×).

**Figure 2:**
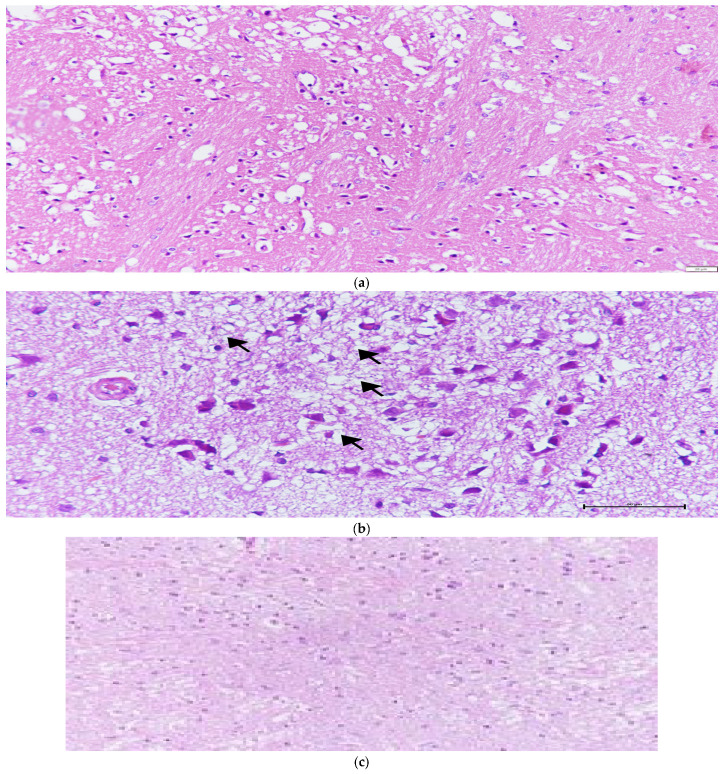
(**a**) Characteristic histopathological features of ischemia-reperfusion injury, including increased eosinophilia, pyknotic degeneration, nucleus shrinkage, vacuolization, necrosis, and edema in the I/R group. (**b**) Vacuolar degeneration and eosinophilic cells in the NAC group; the arrows indicate areas of vacuolar degeneration. (**c**) Edema and vacuolization in the Melatonin + NAC group.

**Figure 3:**
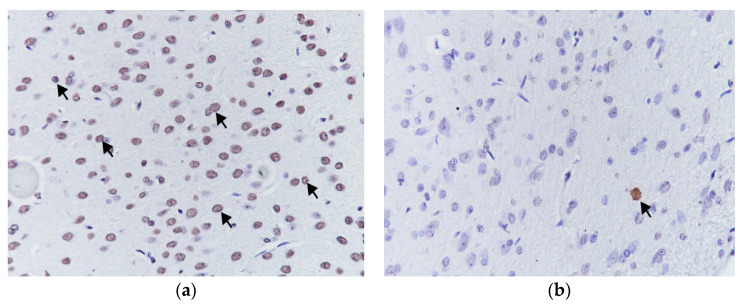
Apoptotic cells showing high (**a**) and low (**b**) reactivity at TUNEL staining, (TUNEL 400×).

Histopathologically, the groups were compared in terms of cellular eosinophilic pyknotic degeneration, vascular congestion, vacuolization, necrosis (infarct), and edema scores. Significant differences were determined between the groups in all scores apart from vascular congestion (*p* < 0.05) (Table 1, Figure 1, Figure 2 and Figure 3). At two-way comparisons, all histological scores were significantly higher in the I/R group than in the control group (*p* < 0.05).

**Figure 4:**
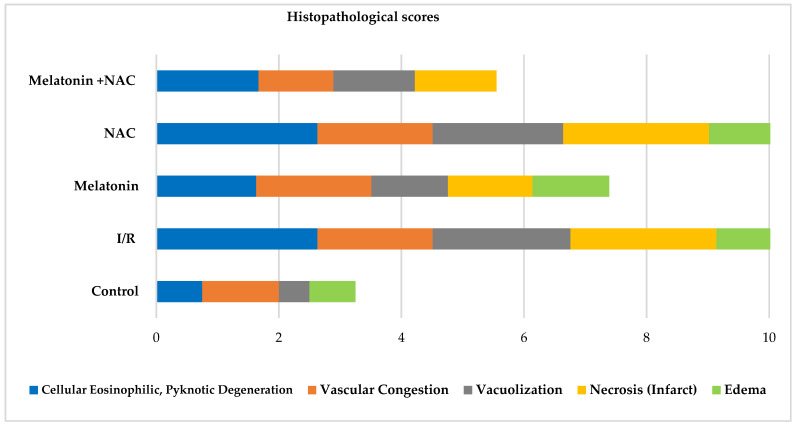
Histopathological scores between groups.

**Figure 5:**
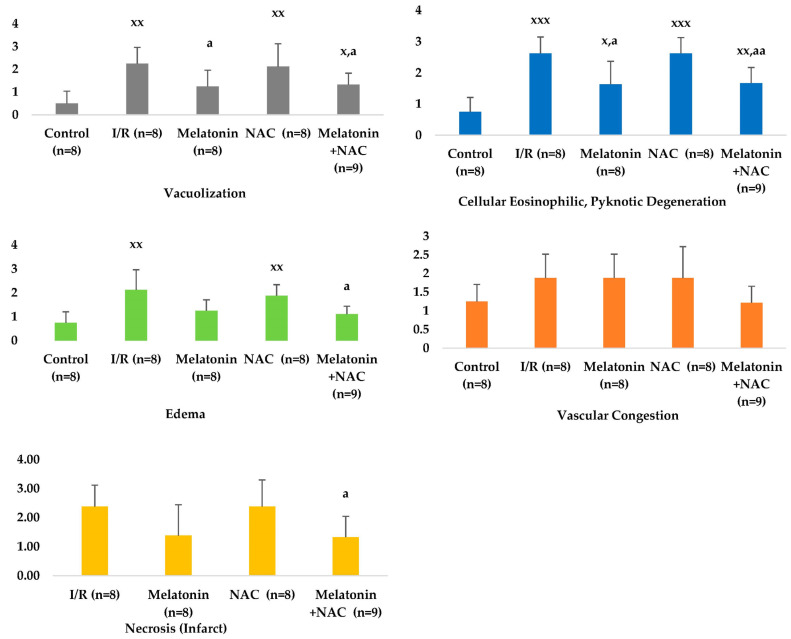
Histopathological scores between groups. x: Comparison with the control group. a: Comparison with the I/R group. x/a *p* < 0.05, xx/aa *p* < 0.01, xxx/aaa *p* < 0.001.

No change occurred in the vascular congestion scores with the administration of melatonin, although decreases were determined in all other scores (Table 2, Figure 4). These decreases were statistically significant for cellular eosinophilic pyknotic degeneration, vacuolization, and edema (*p* < 0.05). No significant change was observed in histopathological scores in the group receiving NAC alone compared to the I/R group (*p* > 0.05). However, all histopathological scores in the group administered NAC together with melatonin were significantly lower than in the MCAO group (*p* < 0.05) (Figure 5).

**Figure 6:**
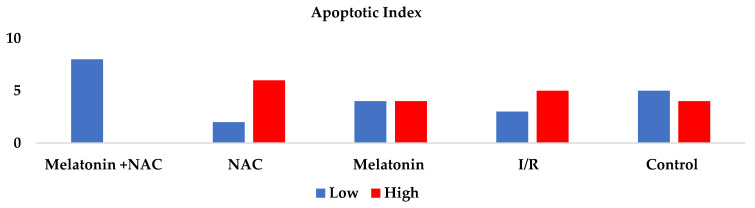
Apoptotic index between groups.

AI was significantly higher in the I/R group than in the control group (*p* < 0.05). Decreases were observed in apoptosis rates in all the treatment groups, although these were not statistically significant (*p* > 0.05) (Table 3, Figure 6).

## 4. Discussion

Organ dysfunction accompanying I/R is generally associated with increasing microvascular permeability, interstitial edema, impaired vasoregulation, inflammatory cell infiltration and parenchymal cell dysfunction, apoptosis, and cell necrosis [20].

The protective activities of melatonin against cell death and inflammatory reactions in various animal models and its effects on hypoxic-ischemic injury processes and the mechanisms involved are currently the subject of active investigation [21]. The effects of melatonin administration (between 1 mg/kg and 50 mg/kg) on ischemic injury have been investigated in different global and focal hypoxia-ischemia models. Studies investigating its neuroprotective effects have generally employed I/R brain injury models induced with occlusion of the MCA. Neuroprotective effects have been obtained with the administration of prophylactic administration immediately before I/R (at doses of 5 mg/kg, 15 mg/kg, 50 mg/kg) or commencing a few weeks before I/R (4 mg/kg/day for nine weeks) [22,23]. Similarly, single or repeat melatonin administration at doses between 1.5 mg/kg and 50 mg/kg within 48 h after I/R has been reported to result in significant decreases in cerebral edema and infarct volume, with concomitant neuronal and glial healing [24,25,26,27,28,29,30].

Studies have shown that the administration of melatonin immediately prior to transient cerebral ischemia or at the beginning of reperfusion results in a decrease in the cellular inflammatory response, edema, and neural cellular immune reactivity against NOS, COX2, and myeloperoxidase, and that these changes are effective in reducing and healing I/R damage in experimental animals [31,32].

The application of melatonin in cerebral ischemia models in mice and rats has been found to exhibit protective effects in different regions of the brain, in the spinal cord, the optic nerve, and in gray and white matter [32,33,34]. Moreover, melatonin has been determined to reduce ischemia-related increasing blood-brain barrier permeability [35]. The application of melatonin in an MCA occlusion model has been shown to be effective in ameliorating cerebral edema and motor behavior findings, and to reduce infarct volume and neuronal cell death [23,36,37]. Studies investigating the neuroprotective effect mechanism of melatonin have reported that it affects different pathophysiological processes involved in the development of I/R injury and exhibits antioxidant, anti-inflammatory, and antiapoptotic effects. Melatonin can exhibit either direct or indirect antioxidant activity. It exhibits its direct antioxidant effects by scavenging free radicals (reactive oxygen, reactive nitrogen, etc.), and its indirect antioxidant effects through regulatory effects on the synthesis and activity of antioxidant enzymes, such as glutathione peroxidase (GPx), glutathione reductase (GRd), superoxide dismutase (SOD), and catalase [38,39,40,41,42]. Melatonin reduces oxidative stress, lipid peroxidation, and radical oxygen species formation, while increasing the activities of antioxidant enzymes, such as SOD, GPx, GRd, and catalase and protecting brain tissue and neurons against ischemic injury [43]. Lin et al. showed that melatonin causes a decrease in the cellular inflammatory response involved in I/R injury, reducing the release of NO and the activity of COX-2 and myeloperoxidase [43]. At the same time, its reduction of the expression of adhesion molecules regulated by nuclear factor kappa β (NF-κβ) has been reported to play a role in its anti-inflammatory and neuroprotective effects of melatonin and to prevent neuronal cell death [44,45]. Melatonin has also been shown to exhibit a protective cerebral effect by inducing the upregulation of anti-apoptotic factors such as I Bcl-2 and a decrease in the pro-apoptotic factor Bax in I/R injury [46]. No change in vascular congestion scores occurred in the I/R model group receiving melatonin (ip 15 mg/kg) in the present study compared to the I/R group, but decreases were observed in the other histopathological scores. These decreases were significant in terms of cellular eosinophilic pyknotic degeneration, vacuolization, and edema scores.

NAC is a precursor of L-cysteine widely employed in mucolytic therapy. Clinically, it is known to be beneficial in the treatment of several diseases, including acetaminophen intoxication, pulmonary oxygen toxicity, certain psychiatric disorders, and human immunodeficiency virus [47,48]. NAC is capable of entering the central nervous system in effective concentrations by passing the blood-brain barrier, and has been shown to be effective in traumatic nervous system injury and age-related neurodegenerative diseases [49,50,51,52]. Studies have also revealed that NAC reduces injury arising following experimental focal and global cerebral ischemia and exhibits neuroprotective effects [53]. This effect of NAC in experimental cerebral ischemia and I/R models has been shown in different regions such as the cortex, hippocampus, and striatum [15,53].

Studies have reported improvement in microcirculation and tissue oxygenation with NAC administration, and that it exhibits preventive and ameliorating effects against brain damage [47,53,54]. NAC has also been shown to exhibit inflammation-lowering effects in ischemia [53]. Researchers have reported that NAC lowers cerebral edema in I/R injury, causes a decrease in the infarct area and number of damaged neurons, and produces improvement in neurological scores [15,55,56]. These neuroprotective effects in I/R injury have been linked to NAC-associated antioxidant effects, such as increased glutathione levels, decreased apoptotic cell death, TNFa, IL-1B, and iNOS expression, and the inhibition of NF-κβ activity [53]. In contrast to the previous literature, no significant change in histopathological scores was observed in the group receiving NAC alone compared to the I/R group in the present study.

Studies have also applied potentially effective drugs against I/R injury together and then evaluated their effects. Danduga et al. showed that NAC and aspirin together exhibited a neuroprotective effect in I/R injury [57]. Liu et al. assessed the effectiveness of combined NAC and normobaric hyperoxia therapy in a transient focal cerebral ischemia model in rats. A combination of NAC and normobaric hyperoxia therapy was found to be more effective than NAC alone in reducing damage [58]. Although studies have evaluated the effectiveness of melatonin and NAC together, we encountered no studies comparing the efficacy of melatonin and NAC in an MCA occlusion-induced model of I/R. Although melatonin and NAC have been reported to exhibit similar efficacy in humans undergoing coronary bypass grafting and to be potent antioxidants, Şener et al. showed that melatonin is more effective than NAC following hepatic I/R injury in rats, and that the effectiveness is significantly increased by creating a synergistic effect with combined treatment [59,60]. Kekeç et al. also reported that melatonin application prevented injury better than NAC after CO intoxication, but that both exhibited individual protective effects [61]. In their study of hypoxic ischemic rats, Wang et al. reported a decrease in brain damage with melatonin at a low dose (5 mg/kg) of melatonin and high-dose NAC (200 mg/kg), but that high-dose melatonin (20 mg/kg) and low-dose NAC (25 mg/kg) produced no neuroprotective effect, and suggested that the effect may be related to the dosages applied [62]. In the present study, melatonin exhibited a greater I/R injury-preventing effect in terms of histological scores and AI values than NAC. When NAC and melatonin were applied together, significant decreases were observed in all histopathological scores compared to the I/R group. Comparison of AI values revealed a decrease in the high apoptotic scores in the treatment groups, although this was not statistically significant. In addition, vascular congestion and edema scores were lower in the group in which NAC and melatonin were applied together compared to the group given melatonin alone. The protective and synergistic effect observed with the combined administration of NAC and melatonin in various studies was also determined in the present research.

## 5. Conclusions

The present study investigated the effectiveness of melatonin and NAC in an I/R injury model induced with occlusion of the MCA. The combined administration of NAC and melatonin was found to exhibit a synergistic effect in preventing I/R injury.

The principal limitation of this study is that only histopathological scoring was employed as a parameter reflecting damage at the cellular level and revealing variation associated with the degree of I/R injury. Although this scoring is recognized as a scientifically valid method for reflecting I/R injury, the biochemical investigation of antioxidant and anti-inflammatory parameters would have strengthened the research. Another limitation of the study is that it was not confirmed by other additional tests. It would also be useful to investigate different doses of NAC and melatonin. Due to budgetary limitations, only a small number of animals was used in this study, so no sham group could be studied and neurobehavioral tests could not be applied.

Although several effective drugs in experimental cerebral ischemia and I/R injury have been described, clinical research to date is insufficient, and no effective therapeutic strategy has yet been developed [63]. Previous research has compared melatonin with other drugs in an MCA occlusion model [64]. The combined use of NAC and melatonin, the neuroprotective efficacy of which on histopathological parameters has been shown in this study, now needs to be supported by further research.

## Figures and Tables

**Table 1:** General characteristics of the study groups.

Group	Name	Surgical Procedure	Medical Procedure
1	Control (*n* = 8)	None	Vehicle (SF + ethanol)
2	MCAO (*n* = 8)	MCAO ----> Reperfusion	Vehicle (SF + ethanol)
3	Melatonin (*n* = 8)	MCAO ----> Reperfusion	Melatonin (15 mg/kg)
4	NAC (*n* = 8)	MCAO ----> Reperfusion	NAC (50 mg/kg)
5	Melatonin + NAC (*n* = 9)	MCAO ----> Reperfusion	Melatonin (15 mg/kg) + NAC (50 mg/kg)

**Table 2:** A comparison of histopathological scores between the study groups.

	Cellular Eosinophilic, Pyknotic Degeneration (Mean ± SD)	Vascular Congestion (Mean ± SD)	Vacuolization (Mean ± SD)	Necrosis (Infarct) (Mean ± SD)	Edema (Mean ± SD)
Control (*n* = 8)	0.75 ± 0.46	1.25 ± 0.46	0.50 ± 0.54	0.00 ± 0.00	0.75 ± 0.46
I/R (*n* = 8)	2.63 ± 0.52	1.88 ± 0.64	2.25 ± 0.71	2.38 ± 0.74	2.13 ± 0.84
Melatonin (*n* = 8)	1.63 ± 0.74	1.88 ± 0.64	1.25 ± 0.71	1.38 ± 1.06	1.25 ± 0.46
NAC (*n* = 8)	2.63 ± 0.502	1.88 ± 0.84	2.13 ± 0.99	2.38 ± 0.92	1.88 ± 0.64
Melatonin +NAC (*n* = 9)	1.67 ± 0.50	1.22 ± 0.44	1.33 ± 0.50	1.33 ± 0.71	1.11 ± 0.33

**Table 3:** Apoptotic index scores between the study groups.

Group	Apoptotic Index (TUNEL) Low/High (*n*)
Control (*n* = 8)	8/0
I/R (*n* = 8)	2/6
Melatonin (*n* = 8)	4/4
NAC (*n* = 8)	3/5
Melatonin + NAC (*n* = 9)	5/4

## Data Availability

The data presented in this study are available on request from the corresponding author.
